# Management of intraocular pressure and inflammation using febuxostat film: *in vitro* - *in vivo* correlation

**DOI:** 10.5599/admet.2601

**Published:** 2025-01-23

**Authors:** Mouli Das, Sk Habibullah, Tanisha Das, Rakesh Swain, Subrata Mallick

**Affiliations:** Department of Pharmaceutics, School of Pharmaceutical Sciences, Siksha ‘O’ Anusandhan (Deemed to be University) Bhubaneswar, Odisha-751003, India

**Keywords:** Anti-inflammation, hydrogel film, plasticizer, *in vitro* release, *in silico* docking

## Abstract

**Background and purpose:**

Urate crystal accumulation may lead to the condition of ocular tophaceous gout, causing ocular inflammation and increased intraocular pressure (IOP) due to the triggering of several inflammatory receptors like NLRP3, A2A, and TLR4. The study has been undertaken to manage intraocular pressure and inflammation using febuxostat (FBX) film formulation for sustained and improved activity, particularly for long-term tophaceous gout patients.

**Experimental approach:**

Hydroxypropyl methylcellulose K100 matrix-based hydrogel film of FBX has been fabricated in the presence of plasticizers like triethanolamine, dimethyl-sulphoxide (DMSO), or polyethylene glycol 600 using casting and evaporation technique. Carrageenan was injected into the upper palpebral region to induce ocular inflammation, and a normotensive rabbit eye model was used for monitoring IOP.

**Key results:**

Amorphization of the drug was observed from the differential scanning calorimetry and X-ray diffraction results. *In vitro* release study revealed an improved and diffusion-controlled sustained drug release for more than 5 h (62.69 to 84.76 %). Compared to its absence, decreased IOP was extended up to 5 h using film (with DMSO). Disappearance of ocular inflammation was also observed in the test animals after 2.5 h of film application, whereas acute inflammation was continued in the group without treatment for more than 4 h. Docking study revealed good binding interaction of drug and NLRP3, A2A, and TLR4 receptor.

**Conclusion:**

Febuxostat-loaded hydrogel-forming plasticized film could be utilized to better manage and control ocular inflammation and associated IOP, particularly in ocular tophaceous gout patients.

## Introduction

Febuxostat is a selective, non-purine analog, xanthine oxidase inhibitor typically used for the treatment of hyperuricemia in patients with gout by reducing uric acid. Xanthine oxidase inhibitors also can control inflammatory reactions [1]. Cisplatin-induced renal inflammation, hepatic and pulmonary inflammation, and also vascular inflammation are known to be diminished by reducing the proinflammatory cytokines TNF-α, IKK-β, or NF-κBp65 after oral administration of febuxostat [2-5]. Chemokine MCP-1, and COX-2 can also be inhibited by xanthine oxidase inhibitors [6-8]. Febuxostat also diminishes inflammatory bowel disease by inhibiting proinflammatory cytokines.

Ocular tophaceous gout is a manifestation of gout in which monosodium urate (MSU) crystals deposit on ocular tissues, which may trigger a strong inflammatory response mediated by NLRP3 inflammasome and proinflammatory cytokines. Chronic inflammation may also damage ocular tissues and lead to complications such as uveitis, keratitis, and scleritis. Deposition of MSU in the anterior part of the eye physically obstructs aqueous humor outflow, leading to an increase in intraocular pressure. The Adenosine A2A receptor, located on the photoreceptor, maintains the release of proinflammatory cytokines by activating the G-protein-coupled receptor [9]. A2A antagonists are acknowledged to prevent oxidative stress, inflammation in glaucoma [10], and age-related macular degeneration [11]. Disruption of the ocular surface barrier leads to an increase in Toll-like receptor 4 (TLR4) expression and is identified for triggering inflammation [12,13].

The major limitation associated with conventional eye drops is the corneal drainage of a drug, resulting in poor bioavailability and frequent administration [14], which could be overcome by using hydrogel-forming ocular film. The application of film is supposed to increase the residence time and, consequently, improve ocular bioavailability [15,16]. Hydrophilic polymer hydroxypropyl methylcellulose (HPMC) has high potency to deliver the drug to wet surfaces where it swells and delivers the drug either by diffusion or controlled release along with mucoadhesive property [16-19].

Hydrogel forming HPMC matrix ocular film of febuxostat has been prepared using triethanolamine (TEA), poly(ethylene glycol) 600 (PEG), and dimethyl sulfoxide (DMSO) as plasticizers. Ocular hypotensive activity and anti-inflammatory potential were also carried out using normotensive rabbit eye and carrageenan-induced rabbit eye models, respectively.

## Materials and method

### Materials

Febuxostat was obtained from Dasami Lab Private Limited (Telangana, India). Hydroxypropyl methylcellulose K100M (HPMC) was purchased from HiMedia Laboratories Pvt. Ltd., Mumbai, India. The plasticizer, TEA, PEG and DMSO were purchased from Burgoyne Co (Mumbai, India), Steer Pharma Lab, (Bangalore, India) and Sisco Research Lab Pvt. Ltd, (Mumbai, India) respectively. All the other reagents used in the experimental work were of analytical grade.

### Methods

#### Hydrogel film preparation of febuxostat

Febuxostat hydrogel film was prepared using HPMC as a matrix-forming polymer by applying the solvent casting method. An amount of 1000 mg of HPMC was dispersed in 40 ml of distilled water in a beaker and left to swell for 24 hours at 4-8 °C. API was solubilized in ethanol along with the respective plasticizer (20 % w/w of HPMC) as tabulated in Table 1. This drug solution was added to the HPMC matrix with continuous stirring in a magnetic stirrer (30-60 rpm) for several hours at room temperature until a clear, homogenous, thick-liquid form was achieved. The prepared thick liquid was poured into a Petri dish and dried at 40 °C to maintain its constant weight. Dried films were separated from the Petri dish and preserved in an air-tight container for the next evaluation.

**Table 1.** Hydrogel forming film formulation of febuxostat using plasticizer (HPMC polymer, ethanol-water solvent system)

Film	Plasticizer (Content, %)
FNA	Nil
FPG	PEG (20)
FDM	DMSO (20)
FTE	TEA (20)

For primary evaluation, the thickness was determined using a digital micrometer (Mitutoyo, Japan), and folding endurance was determined by repeatedly folding a place of the film until it broke. The pH of the films was determined by a pH meter while a piece of film was moistened with distilled water for 10 minutes and the surface pH was observed.

#### Moisture uptake and moisture content

A small portion of the film (about 2×2 cm) was accurately weighed (*W*_1_) and the same was placed over activated silica in a desiccator for 24 hours or more until it gave a constant weight of *W*_2_ (final weight). The moisture content of the film was estimated by using Equation (1):



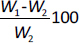

(1)


where *W*_1_ is the initial weight and *W*_2_ is the final weight. The investigation of moisture uptake was determined by placing the dried film (initial weight) into a closed desiccator with 75 % relative humidity maintained with a supersaturated NaCl solution. A constant weight was attained after achieving equilibrium as the final weight and moisture uptake were determined by using Equation (2):



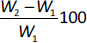

(2)


#### FTIR spectroscopy study

The FTIR study was conducted to evaluate potential interactions between the drug and other constituents of the formulated films. The analysis was carried out using an FTIR spectrophotometer (JASCO FT/IR-4100, Japan) across a wave frequency range of 600 to 4000 cm^-1^.

#### Differential scanning calorimetry

To confirm inertness between the excipients and API, the entire differential scanning calorimetry (DSC) process (DSC-1, Mettler Toledo) was conducted in a nitrogen environment with a heating rate of 10 °C/min and the temperature range was set at 30 to 300 °C.

#### X-ray diffraction

Employing a Cu X-ray supply emission range of 1.5406, the powder XRD (Rigaku, Ultima IV) was utilized to capture the febuxostat film diffraction pattern. The diffraction (2*θ*) in the 5-50° range was recorded at 1° per minute scan speed.

#### Scanning electronic microscopy

Photomicrographs were captured utilizing a gold-coated composition for scanning electronic microscopy (SEM) examination applying 5-15 kV (ZEISS, GEMINI SEM 300)

#### Swelling index

Accurately weighed a small piece of film (*W*_dry_) of size (about 2×2 cm), placed on a glass slide within a petri dish containing pH 6.8 buffer at room temperature. The side holding the sample was taken out and reweighed (*W*_swollen_) at certain intervals (like 60, 90, 120, 180, 240 and 300 minutes) after being carefully swabbed with tissue paper to remove extra fluid from the swelled film surface. This procedure was done in triplicate for individual film formulation to determine the mean*±*SD. Equation (3) was used to determine the swelling index, %:



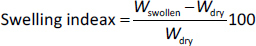

(3)


#### *In vitro* drug release study

The release investigation was conducted using the USP II (Electrolab, dissolution tester USP, TDT06L) dissolution device. The volume of 200 ml of pH 6.8 phosphate buffer is made with potassium dihydrogen phosphate and sodium hydrogen phosphate (Merck Life Science Private Limited). *In vitro* release study carried out at 50 rpm at 34*±*0.2 °C. An accurately weighed piece of film (about 70 to 80 mg) was pasted on top of the glass slide using cyanoacrylate adhesive and kept at the bottom of the dissolution vessel. At regular time intervals of 30, 60, 90, 120, 180, 240, and 300 min, 10 ml of sample was withdrawn. To replenish, the same amount of fresh media was used. Samples were analyzed in a UV spectrophotometer (Shimadzu, 1900i, Japan) at 315 nm. After that, Febuxostat concentration was determined.

#### Kinetics study of film formulation

To examine the release mechanism of the drug from the film, Higuchi, Korsmeyer-Pappas, and Pappas-Sahlin models were implicated in *in vitro* drug release data. Higuchi's model described the drug release kinetics by calculating the linear relationship between cumulative percent drug release and the square root of the time plot. The Pappas-Shalin model makes it possible to assess the mathematical modeling (M) using the constants *k*_1_ and *k*_2_. Fickian diffusion governs the drug release if *k*_1_ > *k*_2_.

Higuchi model is represented by Equation (4):



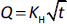

(4)


*Q* is the cumulative amount of drug release at time *t* from the unit area of the film; *K*_H_ is the Higuchi constant.

Korsmayer-Pappas model is represented by Equation (5):



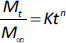

(5)


*M_t_* is the amount of drug released at time *t*; *M_∞_* is the total amount of drug released; *K* is the Pappas release constant; *n* is the release exponent.

Pappas Shalin model model is represented by Equation (6):



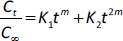



*C_t_*/*C*_∞_ is the fraction dissolved; *K*_1_ and *K*_2_ are Fickian kinetics constant and case II relaxation kinetic constant, respectively and *m* is the diffusion exponent.

### Intraocular pressure

Two groups containing three rabbits were taken to measure the intraocular pressure (IOP) of the febuxostat film formulation. Two drops of proparacaine hydrochloride USP (0.5 % w/v) were instilled in the eye as local anesthesia before the study (within 5 minutes). Group I was the control group without any application of febuxostat. In another group (Group II), considered a test group, febuxostat film formulation containing approximately 10-15 mg (containing about 1.5 to 2 mg of API) was applied topically in the Cul de sac of a rabbit eye. Before applying the hydrogel, it was sterilized using UV radiation for 10 minutes at 25 cm above. Schiotz Tonometer (Riester, Germany) was used to determine the ocular pressure at several time intervals (15, 30, 45, 60, 90, 120, 180, and 240 minutes) after the application of the film. All the measurements were done three times to calculate standard error. After the completion of the study, the rabbit eye was wiped with sterilized saline water, and for fast recovery, MOXI TOR (Moxifloxacin HCl 0.5 % eye drop IP) was instilled.

#### Correlation study

The correlation study has examined the relationship between (a) *in vitro* release *vs.* swelling and (b) *in vitro* release *vs.* area under the decreased IOP curve.

#### *In vivo* anti-inflammatory study

Three groups (containing three male New Zealand rabbits in each group) were taken for anti-inflammatory investigation. Carrageenan injection (200 μl; 2 % w/v) was injected into the sub-conjunctival area of the eye and after an hour of injection, one sterilized film piece was put topically in the Cul-de-sac of the test group (Group I). Only an injection of carrageenan was used to induce in Group II (+ve control). Group III consisted of healthy, normal rabbits that received no therapy. In this investigation, proparacaine hydrochloride was injected for local anesthesia. Finally, moxifloxacin HCl 0.5 % eye drops were utilized for a speedy recovery.

#### Docking study

Computational analysis was done using the Pyrex and Biovia Discovery Studio (version 21.1.0.20298). X-ray crystal structures of receptors were retrieved from the UniProt and Protein DataBank and ligand (febuxostat) were downloaded from the PubChem database in 3D SDF format. The active site of the receptor was predicted from the PrankWeb database. Docking analysis was carried out between Febuxostat with TLR4, A2A, and NLRP3 receptors using Pyrex software. The unit kJ/mol is used to measure the optimum binding affinities of the complex to the protein.

## Results and discussion

### Physico-chemical characterization of febuxostat film

The physical characteristics of febuxostat ocular films are listed in Table 2. The thickness of febuxostat films was between 0.138*±*0.016 and 0.166*±*0.02 mm. Folding endurance was efficient for ocular application, which is >200 and is considered flexible. Surface pH was observed between 7.12*±*0.3 and 7.4*±*0.2, which is compatible with the ocular pH. Moisture content varied from 5.3*±*0.2 to 6.2*±*0.7, while the moisture uptake at a relative humidity of 95 % (RH 95 %) was observed at 13.6*±*0.5 to 24.1*±*0.4. Similar types of physicochemical parameters were observed in previously prepared films for ocular administration [17,20].

**Table 2.** Physical properties of plasticized febuxostat film formulation

Film	Thickness, μm (mean*±*SD)	Surface pH	Folding endurance	Moisture content, %	Moisture uptake, %		
					RH 65 %	RH 75 %	RH 95 %
FNA	158±28	7.1±0.3	>200	5.3±0.2	8.3±0.5	12.4±0.5	13.6±0.5
FPG	150±32	7.2±0.2	>200	6.2±0.7	14.8±0.3	15.6±0.3	16.5±0.4
FDM	138±16	7.4±0.2	>200	6.4±0.2	16.4±0.4	18.2±0.4	18.7±0.4
FTE	166±20	7.3±0.1	>200	6.5±0.3	18.4±1.0	23.2±0.3	24.1±0.4

**Figure 1.**
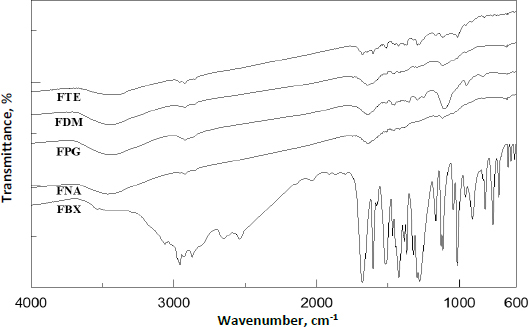
FTIR spectroscopy of pure FBX and prepared ocular films

FTIR spectra of febuxostat and corresponding formulations are portrayed in Figure 1. Potential drug-excipient interaction was examined using FTIR. Febuxostat (FBX) contains a cyanide functional group, a carboxylic acid group, and a thiazole ring. The characteristic peaks of FBX were detected at 2959.23 cm^-1^ due to O-H stretching; nitrile C≡N Stretching appeared at 2228.34 cm^-1^; C=N thiazole stretching was observed at 1519.63 and 1680.66 cm^-1^ appeared due to C=O stretching [21,22]. In prepared film, shifting of C=N stretching at 1509.03 cm^-1^ indicates the presence of interaction between the drug and polymer matrix. The absence of O-H stretching in film formulations may be because of the lack of formation of hydrogen bonds. The broadening of the peak at 1680.66 cm^-1^ indicates a weak formation of van der Walls force or ionic interaction among the drug and other excipients.

**Figure 2.**
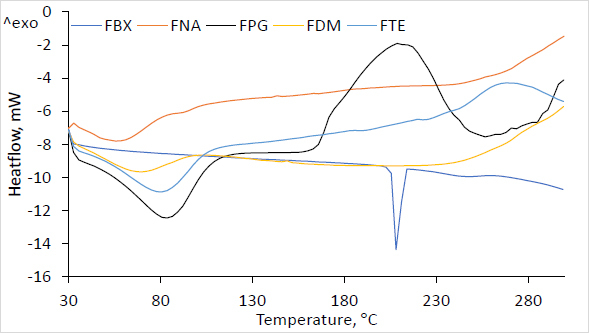
DSC thermogram of pure FBX and prepared ocular film with plasticizer and without plasticizer

In the DSC thermogram, despite any phase transition, the pure FBX’s melting endothermic peak was seen at 208.80 °C [23,24]. However, the absence of strong peaks in the film formulation (Figure 2) suggests that FBX is uniformly distributed throughout the polymer matrix. Water evaporation from the matrix caused the broadening of the peak in a wide range of 50 to 100 °C in the prepared film formulation.

**Figure 3.**
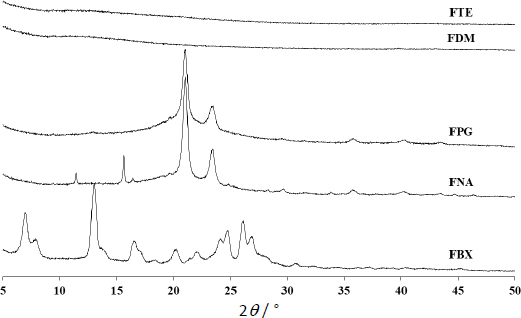
XRD pattern of FBX crystalline drug, plasticised film (FPG, FDM, FTE) and non-plasticised film (FNA) formulation

X-ray diffraction in Figure 3 demonstrates the prepared film’s diffractogram with HPMC, with and without several plasticizing agents, and pure FBX. The crystalline structure was confirmed by the diffraction pattern of FBX, which was detected at 6.98, 13.74, 24.27 and 26.804 at higher intensity [25]. Plasticized films appeared more amorphous than FNA (film without any plasticizer). On the other hand, the lack of diffraction peaks in prepared ocular films of FBX verifies the amorphous form with a distinctive halo pattern.

**Figure 4.**
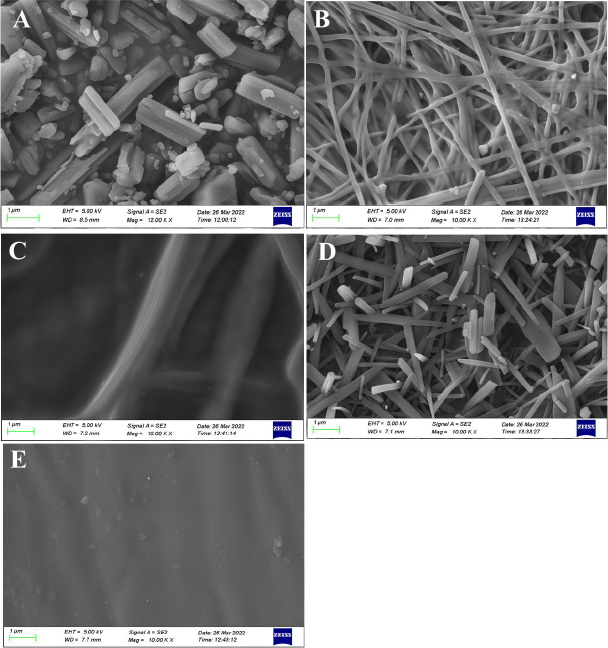
SEM of pure FBX (A); FNA (B); FPG (C); FDM (D); FTE (E)

SEM images were captured and displayed in Figure 4 to examine the surface morphology of any particle or formulation.

The film formulations have completely altered the crystalline structure of raw FBX. Nano-sized crystals have been generated in the case of formulation FDM, and some nanofibers have been discovered in the case of formulation FNA. The drug’s reaction with the plasticizers may cause this type of morphological formation. This type of surface topography may be due to the concentration of nanoparticles even under high shear force mixing conditions polymeric dispersion [26]. The FTE formulation has no visible particles or crystal or fiber formations.

**Figure 5.**
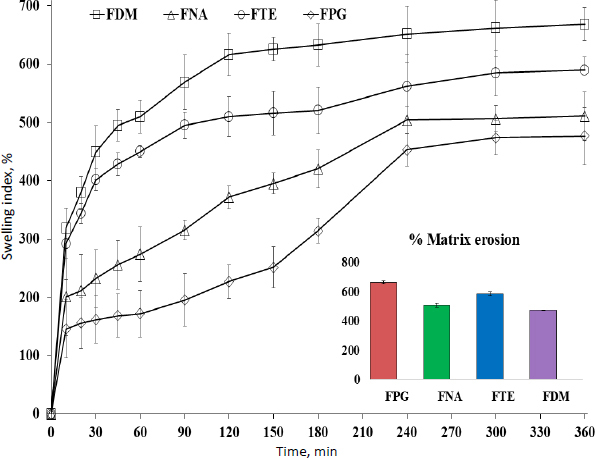
Swelling index (mean*±*SD; *n* = 3) and matrix erosion ((mean*±*SD; *n* = 3) of prepared formulation in HPMC matrix containing plasticizer

Figure 5 shows the level of hydration during 6 hours of swelling. The swelling capacity of polymer is important for its bio-adhesive property to work. Up to a certain threshold, the degree of hydration will boost adhesion; beyond that, untangling at the polymer interface causes a rapid fall in adhesive strength, probably due to erosion. The hydrogel film containing DMSO, *i.e*., FDM, unveiled a maximum swelling rate. The % swelling of the films was exhibited in the order FPG < FNA < FTE < FDM.

### In vitro release study

Chavan *et al.* discussed the use of HPMC polymer to inhibit the crystallization and nucleation of nifedipine. Hydrophilic plasticizers improve the interfacial tension of the polymer matrix. Previous studies suggested an increased and sustained drug release in the presence of PEG [28,29]. Acceleration of drug release was also reported in the presence of DMSO [20,30-32]. TEA was used for better corneal permeability of diclofenac potassium in the HPMC polymer matrix [16].

**Figure 6.**
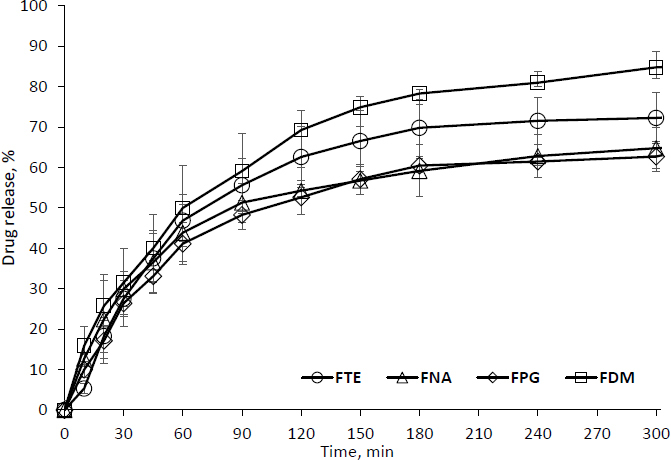
*In vitro* drug dissolution of FBX film formulation

*In vitro* release from film formulations of FBX is depicted in Figure 6. The *in vitro* drug release has been increased to a level in FDM (85.75*±*2.84 % in 5 hours) film, which contains DMSO as a plasticizer, compared to the film that contains no plasticizer (66.06*±*3.62 % in 5 hours). Drug release of 72.25*±*6.22 % was observed in film containing TEA in 5 hours.

**Figure 7.**
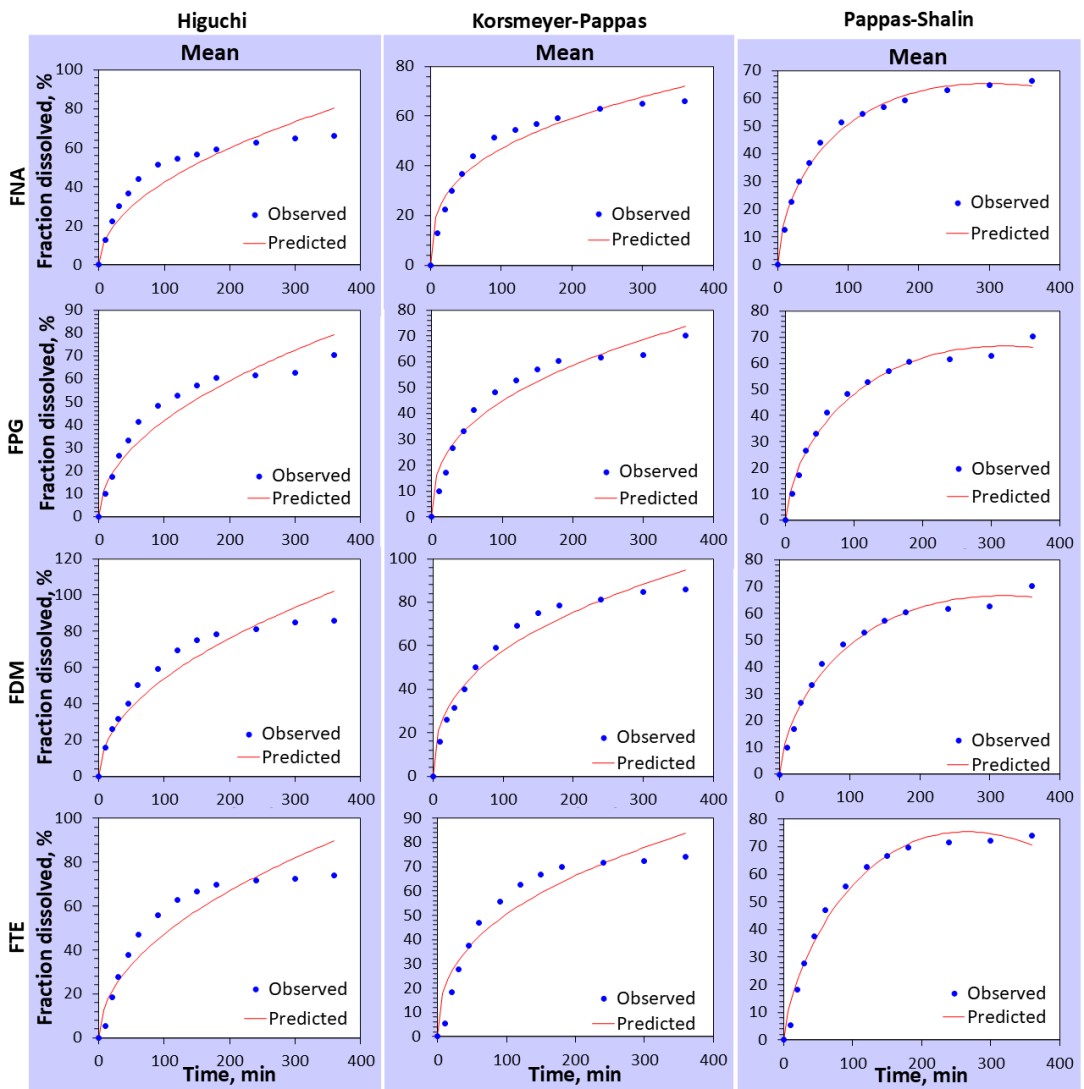
*In vitro* release kinetic models of ocular film

The mechanism of FBX release from the film formulation was estimated using dissolution kinetic models (Higuchi, Korsmeyer-Pappas, and Pappas-Sahlin models). The regression parameters acquired after fitting the release kinetics model to the *in vitro* dissolution are listed in Table 3 and represented in Figure 7. From Higuchi model fitting, all the formulations demonstrated diffusion-controlled drug release where *r*^2^ values were between 0.8627-0.9262. According to the Korsmeyer-Pappas model, the release from the film supports the same diffusion-controlled Fickian pattern where *n* values are less than 0.5 in the absence of any erosion process (*n* = 0.335-0.395). The Peppas-Sahlin model shows that the release pattern follows Fickian diffusion (where *k*_1_ > *k*_2_).

**Table 3.** Diffusional behavior of ocular film using the kinetic model

Formulation code	Higuchi		Korsmeyer-Pappas		Pappas-Sahlin			
	*k*	*r* ^2^	*n*	*r* ^2^	*k*_1_	*k*_2_	*m*	*r* ^2^
FNA	4.242	0.8627	0.335	0.9560	4.567	-0.082	0.588	0.9920
FPG	4.180	0.9187	0.388	0.9545	3.276	-0.040	0.643	0.9862
FDM	5.378	0.9262	0.388	0.9623	3.772	-0.042	0.668	0.9977
FTE	4.729	0.8887	0.395	0.9187	2.587	-0.022	0.729	0.9842

### Correlation study between in vitro release and swelling index

**Figure 8.**
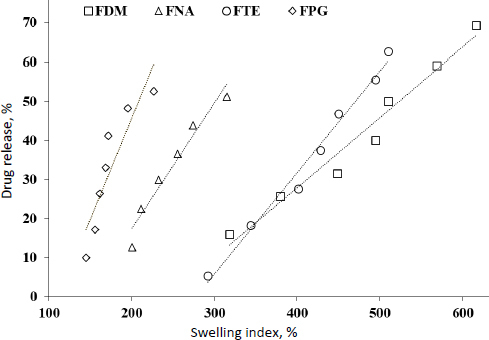
Level A correlation study between *in vitro* drug release and swelling index of FBX hydrogel film

A point-to-point “level A” correlation was detected between *in vitro* drug release *vs.* swelling index (*r*^2^ = 0.820 to 0.985), shown in Figure 8.

### Intraocular pressure

**Figure 9.**
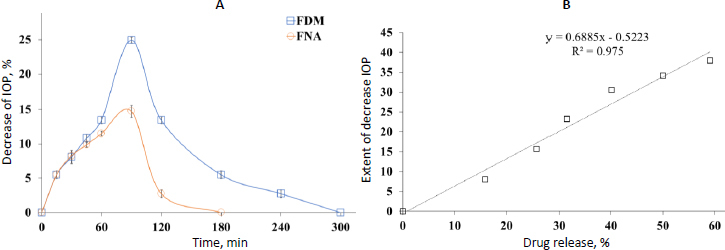
(A) decrease of IOP by administration of film FNA (without plasticizer) and FDM (with DMSO); (B) correlation study: extent of decreased IOP *vs.* drug release

The percentage decrease in intraocular pressure (IOP) against time (min) is represented in Figure 9(A). The decrease of IOP in formulation with DMSO was 24.92*±*0.75 after 90 minutes, whereas the control film formulation showed a mean decreased IOP of 14.72*±*0.88. The lowering of the IOP effect was controlled for 300 minutes compared to the control, which lasted for 180 minutes.

### In vivo anti-inflammatory study

**Figure 10.**
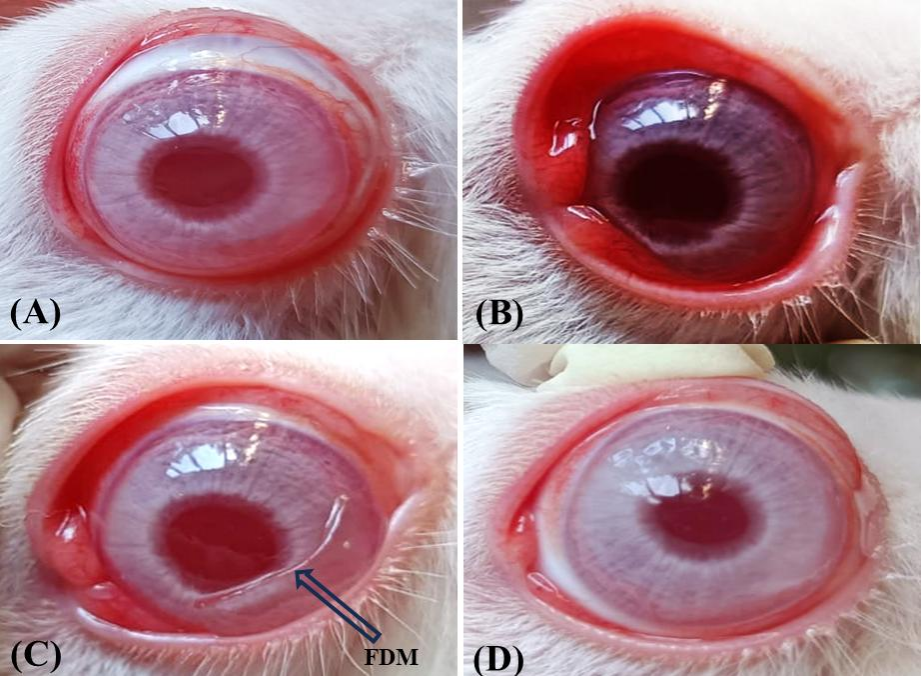
(A) Normal eye before administration of carrageenan; (B) Carrageenan administration; (C) FBX film administration (after 30 minutes) in cul-de-sac area of eye; (D) reduction in redness after 2.5 hours

An anti-inflammatory study was carried out in New Zealand rabbits. Several stages of the anti-inflammatory study are represented in Figure 10. Figure 10 (A) is the normal eye before any induction of carrageenan. Figure 10 (B) represents the redness after carrageenan injection (palpebral region of the eye); Figure 10 (C) administration of FBX film (after 30 minutes) in the Cul-de-sac of the eye; Figure 10 (D) disappearance of tenderness after 2.5 hours of administration of the film.

### Docking study

**Figure 11.**
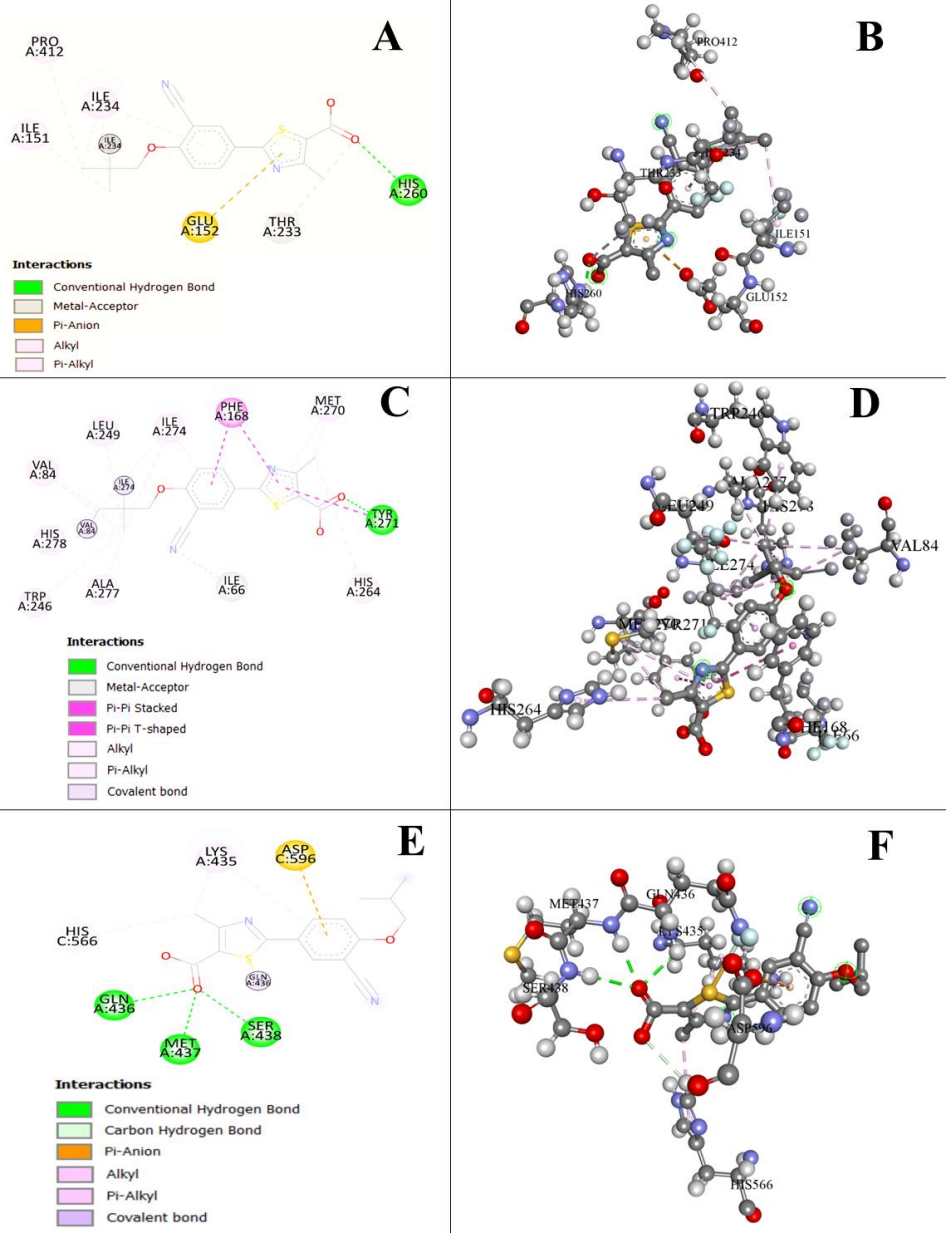
Molecular docking (2D and 3D structures) study of drug with (A, B) NLRP3; (C, D) A2A; (E, F) TLR4

Previous *in-silico* binding studies examined various drugs like minocycline, chloroquine, diclofenac, prednisolone, colchicine, dexamethasone, amlodipine with inflammatory cytokines like IL-1β and the docking score was found -13.095, -16.150, -19.581, -21.882, -22.175, -22.300 and -23.681 kJ/mol respectively [33]. Figure 11 represents the interaction between FBX with several receptors (NLRP3, A2A, and TLR4). These receptors are present in the eye and are responsible for inflammation. It was found that the binding affinity between the drug and the receptors (NLRP3, A2A, TLR4) is very efficient, *i.e*. -35.982, -35.145, and -32.635 kJ/mol, respectively. These docking values clearly showed a greater affinity as compared to the previously reported observations.

## Conclusion

FBX hydrogel film in the HPMC matrix was successfully prepared in the presence of a plasticizer. The amorphization of the prepared plasticized HPMC-FBX film was confirmed by DSC and XRD. The highest % swelling index and low matrix erosion were observed in the film with DMSO (FDM). Maximum *in vitro* drug release (84.76% at 5 h) was observed in FDM with diffusional release kinetics. Normotensive rabbits exhibited decreased intraocular pressure over 5 h after applying film in the Cul-de-sac due to the presence of DMSO rather than its absence (3 h by FNA). A good correlation was observed between the % swelling index and % drug release as well as IOP and % drug release. The inflammation and redness disappeared after 2.5 h of topically applying the film formulation. A good binding affinity was observed with target receptor NLRP3, A2A, and TLR4 of docking score -8.6, -8.4, and -7.8 kcal/mol, respectively, signifying decent biological activity. FBX film formulation could be used to manage IOP and inflammation associated with ocular tophaceous gout.
